# Natural Killer Cells in Liver Disease and Hepatocellular Carcinoma and the NK Cell-Based Immunotherapy

**DOI:** 10.1155/2018/1206737

**Published:** 2018-09-04

**Authors:** Pingyi Liu, Lingling Chen, Haiyan Zhang

**Affiliations:** Laboratory of Department of Hematology, Yishui Central Hospital of Linyi, Linyi, China

## Abstract

Nature killer (NK) cells play a critical role in host innate and adaptive immune defense against viral infections and tumors. NK cells are enriched in liver hematopoietic cells with unique NK repertories and functions to safeguard liver cells against hepatitis virus infection or malignancy transformation. However, accumulating evidences were found that the NK cells were modulated by liver diseases and liver cancers including hepatocellular carcinoma (HCC) and showed impaired functions failing to activate the elimination of the viral-infected cells or tumor cells and were further involved in the pathogenesis of liver injury and inflammation. The full characterization of circulation and intrahepatic NK cell phenotype and function in liver disease and liver cancer has not only provided new insight into the disease pathogenesis but has also discovered new targets for developing new NK cell-based therapeutic strategies. This review will discuss and summarize the NK cell phenotypic and functional changes in liver disease and HCC, and the NK cell-based immunotherapy approaches and progresses for cancers including HCC will also be reviewed.

## 1. Introduction

Liver is a vital organ in human; however, many people suffered from liver disease and liver cancers, such as hepatocellular carcinoma (HCC) which is one of the leading causes of cancer-related death worldwide [1]. The incidence of several major types of cancer, such as lung cancer, colon cancer, and prostate cancer, decreased in recent decade. In contrast, the incidence of HCC increased year by year [1]. In addition, the mortality rate of HCC is similar to the incidence rate which indicates that effective treatments for HCC are lacking in clinic [2, 3]. The major risk factors causing HCC include chronic viral infection, alcohol-related cirrhosis, and nonalcoholic steatohepatitis (NASH) [4]. Chronic hepatitis B virus (HBV) and hepatitis C virus (HCV) infections account for most of HCC cases worldwide [4, 5]; however, NASH will likely become a leading cause of HCC in the future, as the successful HBV vaccination and effective anti-HCV drugs will significantly reduce the number of chronic viral hepatitis patient in the near future [6–8].

In recent decades, accumulating evidences supported that the liver is also an immunological organ with predominant innate immunity [9–11]. The liver is enriched with innate immune cells including Kupffer cells, nature killer (NK) cells, NK T cells, and *γδ* T cells. These cells are critical in host defense against invading pathogens, liver injury and repair, and tumor development [11]. NK cells have been originally described as innate immune cells that are involved in the first line of immune defense against viral infections and tumors. In human, NK cells are phenotypically defined as CD3^−^CD56^+^ large granular lymphocytes. Recently, a population of liver-resident NK cells was defined as CD49a^+^DX5^−^ NK cells in mice. These cells originated from T hepatic hematopoietic progenitors and showed memory-like properties [12, 13]. The counterpart of these liver-resident NK cells was also characterized in human [14, 15]. The functions of NK cells are strictly regulated by the balance of activating receptors and inhibitory receptors interacting with target cells. These receptors can bind to specific ligands; for example, the major histocompatibility complex class (MHC-1) is expressed on healthy hepatocytes, which interacts with inhibitory receptors on NK cells and prevents the activation of NK cells. NK cells can directly eradicate infected cells or tumor cells lacking of MHC-1 molecule expression [16]. Once MHC-1 is downregulated by viral infection or tumorigenesis on the hepatocytes, the NK cells will loss the inhibitory signal controlled by the interaction of the NK inhibitory receptor with the MHC-1 complex, and the NK cells will be activated to kill infected hepatocytes. In the liver, the percentage of NK cells in total lymphocytes is around 5 times higher than the percentage in peripheral blood (PB) or spleen; thus, the NK cells were considered to play a very important role in the prevention of HCC and therefore were considered a potential cell therapy resource for the treatment of HCC [17].

In this review, we will summarize the phenotypes and functions of NK cells in chronic viral hepatitis, alcoholic liver disease, NASH, and HCC, and the progresses in NK cell-based immunotherapy for cancers but not limited to HCC are also reviewed.

## 2. NK Cells in Chronic Viral Hepatitis

Chronic viral hepatitis including HBV and HCV is the leading cause for the development of liver cirrhosis and subsequent HCC. HBV and HCV are pathogen replicate and grow inside of hepatocytes which alter the surface molecule for the interaction with NK cells. NK cells are critical in the early immune response for the clearance of virus. In chronic HBV and HCV patients, the percentage of circulating PB NK cells was lower than that in healthy controls [18–21]. In addition, the production of proinflammatory cytokines such as interferon gamma (IFN-*γ*) and tumor necrosis factor-*α* (TNF-*α*) by NK cells was decreased in chronic HBV and HCV patients [18, 19, 22]. However, the impact of chronic viral infection on the cytolytic activity of NK cells was controversial. Several reports showed that NK cytolytic effector function was not changed [18, 20], while other researchers observed impaired NK cytolytic activity [23–25]. The differences between these studies were probably due to the lack of standardized protocol and reagents as well as the heterogeneity of patients. The phenotype of NK cells between HBV and HCV patients was different, and NK cells showed an increase in the activating receptor NKG2D expression and a decrease in inhibitory receptor expression phenotype in chronic HCV patients, while in chronic HBV patients, the percentage of activating NKG2C^+^ NK cells increased and the inhibitory receptor expression was normal [18]. The reduced percentage and impaired function of NK cells in chronic viral hepatitis patients were believed to contribute to the disease progression and the transformation of HCC.

## 3. NK Cells in Alcoholic Liver Disease

Alcoholic liver disease is caused by alcohol abuse and also considered a major cause for cirrhosis and HCC, and around half of liver cirrhosis was related to alcohol [26]. NK cells were shown to play a critical role in resolving fibrosis by directly killing activated hepatic stellate cells (HSCs) and inducing HSC apoptosis by the production of IFN-*γ* [27]. However, chronic exposure to alcohol reduced the expression of NKG2D, tumor necrosis factor-related apoptosis-inducing ligand (TRAIL), and IFN-*γ* on NK cells, which subsequently abrogated the antifibrotic effects of NK cells [28, 29]. In addition, the suppression of NK cell activation by chronic alcohol consumption was supported by several studies [30–32]. The elevation of NKG2D, granzyme B, perforin, Fas ligand (FasL), TRAIL, and IFN-*γ* expression in NK cells by poly I:C stimulation was blocked in ethanol diet-fed mice [30]. More evidence and further study are needed to confirm whether the impairment of NK cell function by chronic alcohol consumption contributes to the development of alcohol-related HCC.

## 4. NK Cells in NASH

Although the incidence of chronic viral hepatitis decreases benefiting from the vaccinations and the advanced clinical therapies, the incidence of HCC gradually increases in developed countries. The nonalcoholic fatty liver disease (NAFLD) and NASH become two of the leading causes of HCC. However, there were only very limited studies about the role of NK cells in the pathogenesis of NAFLD and NASH. Less circulating PB NK cells and cytotoxic ability were found in patients with obesity than in healthy controls [33]. In contrast, another report showed that NK cell-derived mediators such as NKG2D and TRAIL mRNA levels were increased in NASH patients [34]. In a recent study, DX5^+^ NKp46^+^ NK cells were found to be increased in a NASH mouse model. Moreover, these NKp46^+^ NK cells regulate M1/M2 polarization of liver macrophages and inhibit the development of liver fibrosis in the NASH model [35]. So, how the dysregulation of NK cells in NASH contributes to HCC development is still largely unknown.

## 5. NK Cells and HCC

As we discussed above, NK cells are enriched in healthy liver, and they play critical roles in the surveillance of HCC. Similar to chronic viral hepatitis, the number of peripheral NK cells, especially CD56^dim^CD16^+^ NK cells, dramatically reduced in HCC patients [36, 37]. The NK cell number in the liver tumor area was also less than the NK cell number in the nontumor area and showed impaired cytotoxic ability as well as IFN-*γ* production [36, 38]. Moreover, the number of infiltrating and CD56^+^ NK cells positively correlated with HCC cancer cell apoptosis and patient survival [39, 40]. Several mechanisms had been proposed to explain the defect of the NK cell number and function in HCC; for example, infiltrated monocytes/macrophages in the peritumoral area induced rapid activation of NK cells. These activated NK cells then became exhausted and eventually died which was mediated by the CD48/2B4 interactions [38]. Myeloid-derived suppressor cells (MDSCs) may also interact with NK cells in HCC patients. MDSCs from HCC patients directly inhibited cytotoxicity and cytokine production of NK cells in a contact-dependent manner [41]. In addition, fibroblasts derived from HCC triggered NK cell dysfunction which was mediated by indoleamine 2,3-dioxygenase (IDO) and prostaglandin E2 (PGE2) produced by the HCC cells, and these two natural immunosuppressants downregulate activating NK receptors [42]. Taken together, multiple mechanisms were involved in the NK cell malfunction, resulting in the development of HCC, and the lack of the NK cell number and the defects in function of NK cells facilitated the escape of tumor cells from immune surveillance.

## 6. NK Cell-Based Immunotherapy for Cancer

As we discussed above, NK cells, as a type of innate immune cell, are rapidly mobilized and serve as the first-line immune responders against viral-infected cells and tumor cells; however, the NK cells usually become inhibited in many cancers including HCC. Studies have been performed to explore NK cell-based immunotherapy for cancers. These approaches include either endogenous stimulation of the NK cells in patients, such as administration of cytokines to activate NK cells in cancer patients and treatment of the patients with antibodies that target the NK inhibitor receptor or checkpoint protein to activate NK cells or agonist of NK cell activating receptors, or adoptive NK cell transfer to the cancer patients. The therapeutic effects and progresses of these approaches will be reviewed in this article.

### 6.1. Cytokine Treatment to Increase the Cytotoxicity of NK Cells

Interleukin-2 (IL-2) was the first FDA-approved cytokine to boost cytotoxicity of NK cells [43]. However, IL-2 can act not only on NK cells but also on T cells including regulatory T cells (Tregs) which may inhibit NK cell function. Depletion of Tregs improved NK cell proliferation and outcome in acute myeloid leukemia (AML) therapy [44]. Several strategies were used to improve the efficiency of IL-2, including a modified IL-2 called “super-2” which can activate NK cells and cytotoxic CD8 T cells but not Tregs [45]. The fusion protein of IL-2 and NKG2D ligand only activated on NKG2D-expressing NK cells but on T cells [46].

Interleukin-15 (IL-15) is another cytokine to boost NK activity and increase cytotoxicity of both NK cells and CD8^+^ T cells without activating Tregs [47]. IL-15 treatment significantly increased the number of NK cells in both mouse model and human patients [48, 49]. Several IL-15 fusion proteins or modified IL-15 have been developed to increase their stability and efficiency [50–52]. The clinical trials for the antitumor effect of IL-15, modified IL-15, and IL-15 fusion proteins are ongoing.

### 6.2. Antibodies to Modulate the NK Cytotoxic Function

Killer cell immunoglobulin-like receptors (KIRs) are expressed on NK cells and a minority of T cells [53]. KIRs bind with MHC-1 molecules to control the cytotoxic function of these immune cells. NK cells express both inhibitory KIR and activating KIR. The balance between the NK KIR inhibitory signal and KIR activating signal defines the NK effector function. Tumor cell-derived MHC-1 or class I-like molecules usually bind to KIR to inhibit NK cell activation [54]. Antibodies against inhibitory KIR showed promising effects on promoting NK cell cytotoxicity in mouse models [55]. However, the clinical trial did not show favorable effects of the KIR2D (an activating KIR receptor) antibody IPH2101 in multiple myeloma (MM) patients [56]. The lack of efficiency of the KIR2D antibody IPH2101 was probably due to the depletion of KIR2D-expressing NK cells by the antibody. The combination of lenalidomide, an immunomodulatory drug that can activate NK cells, with IPH2101 showed promising beneficial effects on MM patients in a phase I clinical trial [57].

Another NK receptor target to improve the cytotoxic function of NK cells is NKG2A. NKG2A is expressed in NK and CD8^+^ T cells as an inhibitor receptor. The heterodimer of NKG2A and CD94 can bind to HLA-E which is usually upregulated in tumor cells [54]; therefore, the tumor cells escaped the NK by this inhibitory signal. An antibody targeting NKG2A showed increased NK cell cytotoxicity and reduced disease progression in human primary leukemia or Epstein-Barr virus cell line-infused mouse model [58]. The clinical trial is ongoing to evaluate its safety and efficiency for cancer therapy.

Programmed cell death protein 1 (PD-1) is a well-known immune checkpoint of T cells. It is a cell surface receptor; the integration with its ligand on target cells suppresses the T cell activation and plays an important role in downregulating the immune system. Recently, the PD-1 receptor is found to be highly expressed on PB and tumor-infiltrating NK cells from patients with multiple myeloma (MM) and with digestive cancers including esophageal, colorectal, biliary, gastric, and liver cancers [59]. In the digestive cancer study, the investigators showed that PD-1 upregulation on NK cells correlated with poorer disease outcome in esophageal and liver cancers. In both the MM study and the digestive cancer study, PD-1-blocking antibody could increase the NK cytotoxic function in vitro targeting the tumor cell line or primary cancer cells. Moreover, the PD-1 antibodies inhibited the tumor growth in a xenograft mouse model [59]. These findings suggested that PD-1 blockade might be an efficient strategy in NK cell-based tumor immunotherapy.

In a recent study, Zhang et al. have found that the checkpoint inhibitory receptor TIGIT (“T cell immunoglobulin and immunoreceptor tyrosine-based inhibitory motif domain”) was highly expressed on the exhausted tumor- (colorectal cancer) associated infiltrating NK cells. Monoclonal antibody-mediated blockade alone which targets TIGIT or in combination with the antibody against the PD-1 ligand PD-L1 increased the antitumor activity of both NK cells and T cells in preclinical mouse models and efficiently delays tumor growth [60]. Therapeutic strategies combining multiple blocking antibodies to treat cancer patients with NK cell exhaustion may improve the therapeutic efficacy, although the side effect of the antibody treatment needs to be carefully evaluated.

### 6.3. Agonist of NK Cell Activating Receptors

NK cells express a variety of activating receptors that play critical roles in regulating NK cell function. Major activating receptors on NK cells were well studied such as NKG2D, NKp30, NKp44, NKp46, and NKp80 [61]. Induction of NK cell activation to release cytokines or direct kill target cells requires combinatorial activating receptor synergy [62]. Activating receptor downregulation is observed in cancer patients and was correlated with poor disease outcomes [63–66]. It is believed that the tumor cells express a high soluble amount of activation receptor ligand that induced the downregulation of activation receptors on NK cells in the patients [66–68]. Modulating NK cell activating receptor expression by neutralizing their soluble ligand secreted by tumor cells in patients is a potential clinical therapeutic strategy.

### 6.4. Adoptive Transfer of NK Cells

NK cell adoptive transfer therapy requires the NK ex vivo expansion, in vivo long persistence, maximal in vivo activity, and NK cell killing specificity. The source of NK cells for adoptive transfer therapy can be autologous or allogeneic PB NK cells, stem cell-derived NK cells, and NK cell lines such as NK-92 and KHYG-1. Fresh or expanded NK cells derived from patients (autologous) failed to show improved clinical outcome in several types of cancers [69, 70]. The reason was that these transferred NK cells remained in circulation rather than in tumor tissue [69]. In contrast, NK cells derived from a healthy donor (allogeneic) showed a positive response in AML patients [71], and the following studies also showed encouraging results by using haploidentical NK cells for both elderly and pediatric AML patients [72–74]. The clinical efficacy of the allogeneic NK cell adoptive immunotherapy to treat solid tumors has also been evaluated by several groups [75–77]. Lin et al. had reported that percutaneous cryoablation combined with allogeneic NK adoptive transfer significantly increased the median progression-free survival of advanced HCC patients [75]. And multiple allogeneic NK cell infusion was associated with better prognosis in advanced cancers, including advanced HCC [75] and stage III pancreatic cancer [76]. Allogeneic NK cell immunotherapy for advanced non-small-cell lung cancer also showed a significant benefit in clinic [77]. The successful allogeneic NK cell adoptive transfer therapy may depend on the protocol for isolation and expansion and the purity of NK cells [78–80].

To increase the killing specificity and efficiency of NK cell-based immunotherapy, several genetic modification approaches have been developed. The genetic modification for NK cells was not as easy as that for other immune cells such as T cells due to the resistance to retroviral infection. Several strategies were explored to improve the efficiency for transfection in NK cells [81, 82]. The introduction of CD16a, IL-15, and IL-2 in NK cells may increase the proliferation, activation, and cytotoxicity of NK cells [83–85]. Recently, the successful application of the chimeric antigen receptor (CAR) in T cells was adopted in NK cells to increase the specificity and efficiency of NK cell immunotherapy. Compared with CAR-T cells, CAR-NK cells were short lived, which reduced the risk for autoimmunity and tumor transformation. The cytokines released from NK cells such as IFN-*γ* and granulocyte-macrophage colony-stimulating factor (GM-CSF) were safer than the cytokine storm in CAR-T cell therapy [86]. As we mentioned above, due to the difficulties in expansion and transduction of primary NK cells, the only FDA-approved cell line for use in clinical trials, NK-92, was considered an ideal NK cell source for CAR-NK cell therapy [87–89]. ErbB2/HER2-specific CAR-NK-92 cells showed very encouraging efficiency in target therapy of glioblastoma in an animal model [90]. CD19-specific CAR-NK-92 cells were sufficient to lyse CD19^+^ B-precursor leukemia cell lines as well as lymphoblasts from leukemia patients [91, 92]. In a preclinical study, CD5 CAR-NK-92 cells shows consistent, specific, and potent antitumor activity against T cell leukemia and lymphoma cell lines and primary tumor cells [93]. For the HCC, the first report was published very recently; glypican-3- (GPC3-) specific CAR-NK-92 cells showed potent antitumor activities in multiple HCC xenografts with both high and low GPC3 expressions. As expected, the GPC3 CAR-NK-92 cells did not show cytotoxicity to GFP3-negative HCC cells [94]. Currently, 9 clinical trials are ongoing to evaluate the safety and efficiency of CAR-NK cells, of which 7 trials are used for leukemia or lymphoma and 2 trials are for solid tumor. In the current preclinical study, some other NK sources are under investigation [95], such as the NK cells derived from hematopoietic progenitor cells [96] or from cord blood, and expanded in vitro with the aAPC K562-based system [97].

## 7. Summary

NK cells have been discovered for more than 40 years [98]; however, how to manipulate NK cells for the therapy of diseases remains elusive. The impairment of the function of NK cells was observed in many types of liver diseases including chronic viral hepatitis, alcoholic and nonalcoholic steatohepatitis, and HCC. Several approaches have been developed to boost the activity of NK cells for therapeutic purpose, but most of these studies are still in a preclinical stage. With the success of ex vivo genetic modification of T cells in the therapy of leukemia, it is promising that the similar strategy, as wells as other approaches to regulate the balance between activating and inhibitory receptors in NK cells, might also lead to the successful treatment for various liver diseases including HCC.
